# Out of Tanganyika: Genesis, explosive speciation, key-innovations and phylogeography of the haplochromine cichlid fishes

**DOI:** 10.1186/1471-2148-5-17

**Published:** 2005-02-21

**Authors:** Walter Salzburger, Tanja Mack, Erik Verheyen, Axel Meyer

**Affiliations:** 1 Lehrstuhl für Zoologie und Evolutionsbiologie, Department of Biology, University of Konstanz, 78467 Konstanz, Germany; 2 Vertebrate Department, Royal Belgian Institute of Natural Sciences, 1000 Brussels, Belgium

## Abstract

**Background:**

The adaptive radiations of cichlid fishes in East Africa are well known for their spectacular diversity and their astonishingly fast rates of speciation. About 80% of all 2,500 cichlid species in East Africa, and virtually all cichlid species from Lakes Victoria (~500 species) and Malawi (~1,000 species) are haplochromines. Here, we present the most extensive phylogenetic and phylogeographic analysis so far that includes about 100 species and is based on about 2,000 bp of the mitochondrial DNA.

**Results:**

Our analyses revealed that all haplochromine lineages are ultimately derived from Lake Tanganyika endemics. We find that the three most ancestral lineages of the haplochromines *sensu lato *are relatively species poor, albeit widely distributed in Africa, whereas a fourth newly defined lineage – the 'modern haplochromines' – contains an unparalleled diversity that makes up more than 7% of the worlds' ~25,000 teleost species. The modern haplochromines' ancestor, most likely a riverine generalist, repeatedly gave rise to similar ecomorphs now found in several of the species flocks. Also, the Tanganyikan Tropheini are derived from that riverine ancestor suggesting that they successfully re-colonized Lake Tanganyika and speciated in parallel to an already established cichlid adaptive radiation. In contrast to most other known examples of adaptive radiations, these generalist ancestors were derived from highly diverse and specialized endemics from Lake Tanganyika. A reconstruction of life-history traits revealed that in an ancestral lineage leading to the modern haplochromines the characteristic egg-spots on anal fins of male individuals evolved.

**Conclusion:**

We conclude that Lake Tanganyika is the geographic and genetic cradle of all haplochromine lineages. In the ancestors of the replicate adaptive radiations of the 'modern haplochromines', behavioral (maternal mouthbrooding), morphological (egg-spots) and sexually selected (color polymorphism) key-innovations arose. These might be – together with the ecological opportunity that the habitat diversity of the large lakes provides – responsible for their evolutionary success and their propensity for explosive speciation.

## Background

*"At some stage in the past the waterways of Africa were, from the fishes' point of view, accessibly interconnected." *P. H. Greenwood (1983)

With estimated numbers of about 1,000 and 500 species respectively the assemblages of cichlid fishes of East African lakes Malawi (LM) and Victoria (LV) are, by far, the most species-rich species flocks [1-6]. In Lake Tanganyika (LT), Africa's oldest lake, "only" about 200 to 250 cichlid species occur, but they are phenotypically, behaviorally and genetically more diverse [7-9]. Remarkably, almost 100% of the species of all of these flocks are endemics and the three East African Great Lakes do not have a single cichlid species in common [10]. While in LT at least twelve eco-morphologically distinct tribes can be clearly distinguished phylogenetically [7,9,11], the species flocks of LM and LV are entirely comprised of cichlids assigned to only one of these tribes, the Haplochromini [1,4,10,12]. Additionally, several less species-rich flocks of haplochromines are found in smaller lakes in East Africa [10], some of which have been combined with the LV radiation into a "superflock" of closely related species [4,12,13].

**Figure 1:**
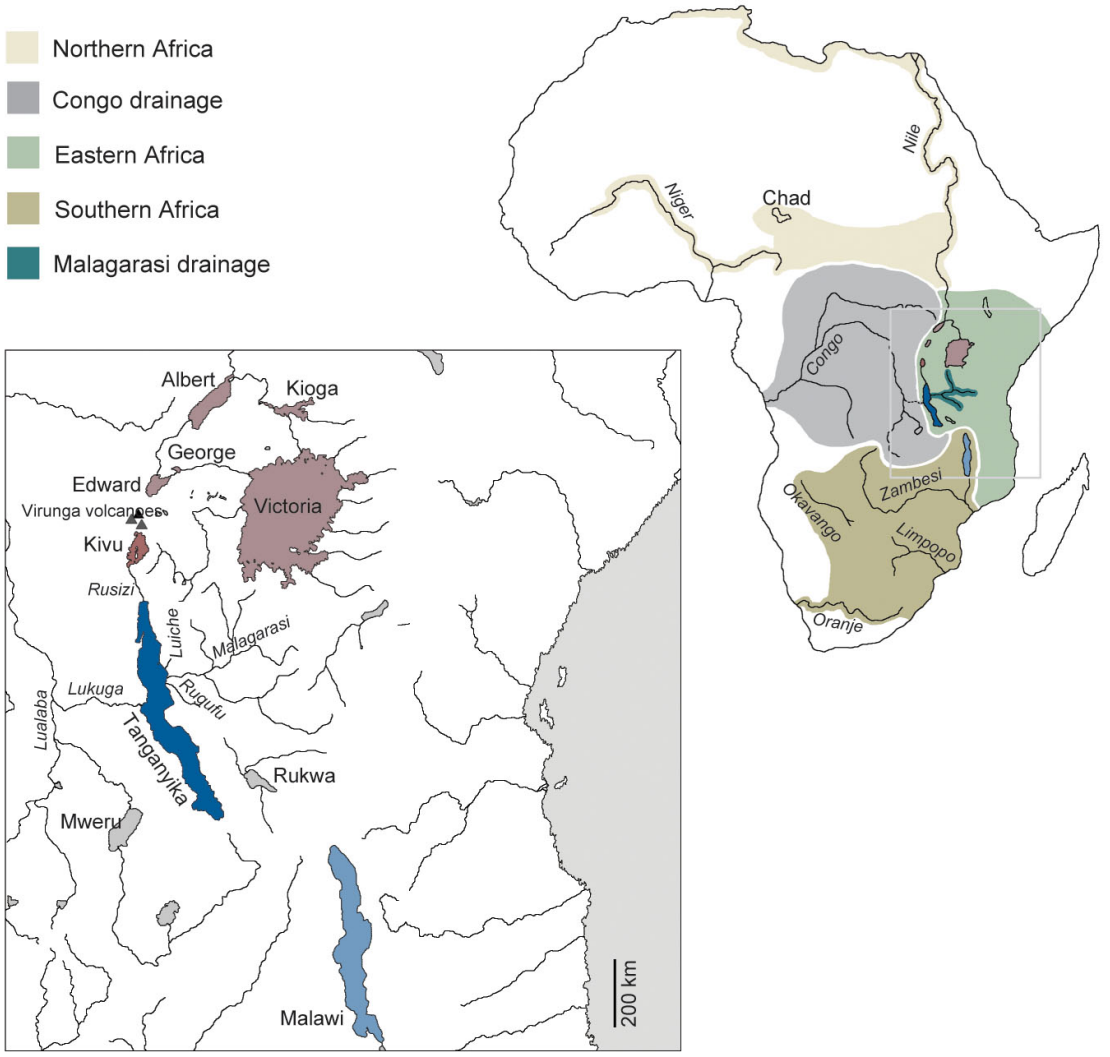
**Distribution of the major haplochromine lineages in Africa with special emphasis on the East African lakes **(according to our phylogenetic and phylogeographic analyses and references [4, 12, 13, 34]). Note that the color scheme is carried throughout this publication.

Not all haplochromines are lacustrine, however, and some 200 species inhabit rivers and occur in northern-, eastern-, southern- and central-Africa but are virtually absent from West Africa [10,12] (Fig. 1). Traditionally, it was believed that riverine haplochromines seeded the cichlid radiations in all East African Great Lakes [8,10]. Molecular-based phylogenies indeed uncovered haplochromine lineages that are ancestral to the species flocks of LM and LV (see *e.g.*, [1,4,9,14]. In contrast, the primary radiation of LT's cichlid assemblage was found to predate the origin of haplochromine lineages [9,15,16] suggesting a close phylogenetic affinity of the ancestor(s) of the haplochromines to other tribes in LT [9]. However, only a relatively small fraction of the genetic and geographic diversity of the haplochromines has been included in previous phylogenetic and phylogeographic studies.

That the haplochromines have a particularly strong propensity for explosive speciation among cichlids is best illustrated by their unparalleled species-richness and diversity. With the exception of two LT lineages (Lamprologini: ~70–100 species; Ectodini: ~25–30 species), discrete adaptive radiations of non-haplochromines are comprised by about a dozen species at most. Thus, which evolutionary novelties might be causally related to the explosive speciation that distinguish the haplochromines from all other cichlid lineages remains a crucial question for the understanding of the explosive patterns of cichlid evolution in East Africa. Haplochromines furthermore represent prime examples for parallel evolution, and it is particularly the lacustrine haplochromine species flocks that independently evolved morphologies and color-patterns that are convergent between species of different lakes [2,17,18].

In order to gain a deeper understanding of the adaptive radiations of cichlids in general and the formation of East African cichlid species flocks in particular, several crucial questions still remain to be answered: (i) What evolutionary lineages from which geographic ranges make up the diversity of haplochromines? (ii) Which were the founding lineages of the lacustrine adaptive radiations and were they riverine generalists? (iii) Can particular behavioral and/or morphological key-innovations be identified that might be causally related with the diversification of haplochromines? With the aim of addressing these questions, we conducted the most extensive phylogenetic and phylogeographic study of haplochromine cichlids so far, analyzing a portion of up to 2,000 bp of the mitochondrial genome of about 100 species. We included representatives of relevant cichlid tribes from LT [7,9,15] as well as members of all major riverine and lacustrine haplochromine lineages and all but one haplochromine genus (following [12]).

## Results

**Figure 2:**
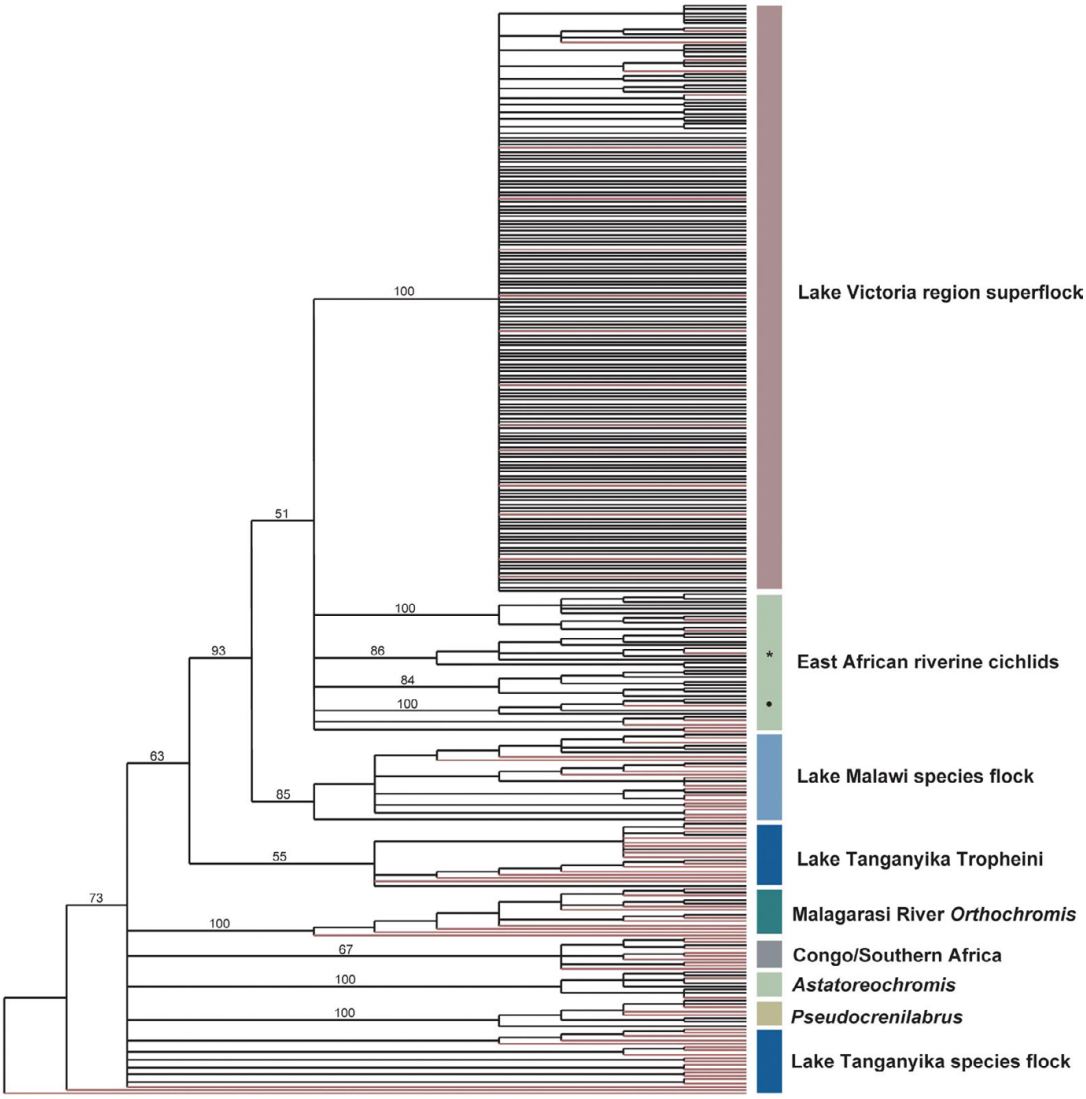
**50% majority-rule bootstrap consensus tree of 304 cichlid fish specimens based on 862 bp of the mitochondrial control region **(neighbor-joining, HKY85 model with gamma substitution correction, 5,000 replicates). Relevant bootstrap values are depicted on the respective branches. The branches colored in red indicate the taxa that were chosen for the phylogenetic analyses combining the control region with sequences of the NADH dehydrogenase subunit II gene (see Fig. 3; for *Ctenochromis oligacanthus *from GenBank no control region sequence was available). The colors of the boxes that indicate the major clades refer to Figs. 1 and 3, the labels of the clades correspond to Table 1 [see Additional file 1]. Note that *Haplochromis bloyeti *(marked by an asterisks) had a control region sequence identical to *H. sp. *1533 of [25], which was collected in the Malagarasi River, and grouped – together with other fishes from the Malagarasi area and from the Lake Edward/George region – into their group VII. Likewise, our *H. sp. *Tanzania I (marked by a circle) was identical to *H. sp. *1738 of [25], which was collected in Lake Chala and clustered with other taxa from Tanzania into their group VI. In addition to *Haplochromis gracilior *(endemic to Lake Kivu), which was recently identified as close relative of the Lake Victoria superflock [4], we found another sister group to the superflock. This lineage includes *Haplochromis paludinosus *that occurs in the Malagarasi, as well as undescribed species from Tanzania and Lake Edward (see also Fig. 3). Like *Haplochromis gracilior *from Lake Kivu, all these taxa have the diagnostic character state 'Adenine' in position 630 of the control region alignment and root to the Lake Victoria superflock through the central rift valley haplotype [4], corroborating the view that Lake Kivu is the main reservoir from which the Lake Victoria superflock evolved [4].

The neighbor-joining analysis based on 304 complete mitochondrial control region sequences (Fig. 2) could not resolve the phylogenetic relationships between haplochromine lineages and the LT lineages with convincing bootstrap support. However, this analysis, which primarily aimed to provide a basis for the selection of taxa for the second set of analyses, already indicated the existence of a monophyletic clade that is comprised by the Tanganyikan Tropheini, sister-group to a clade consisting of the LM cichlids plus several East African riverine and lacustrine lineages and the representatives of the LV region superflock [4].

**Figure 3:**
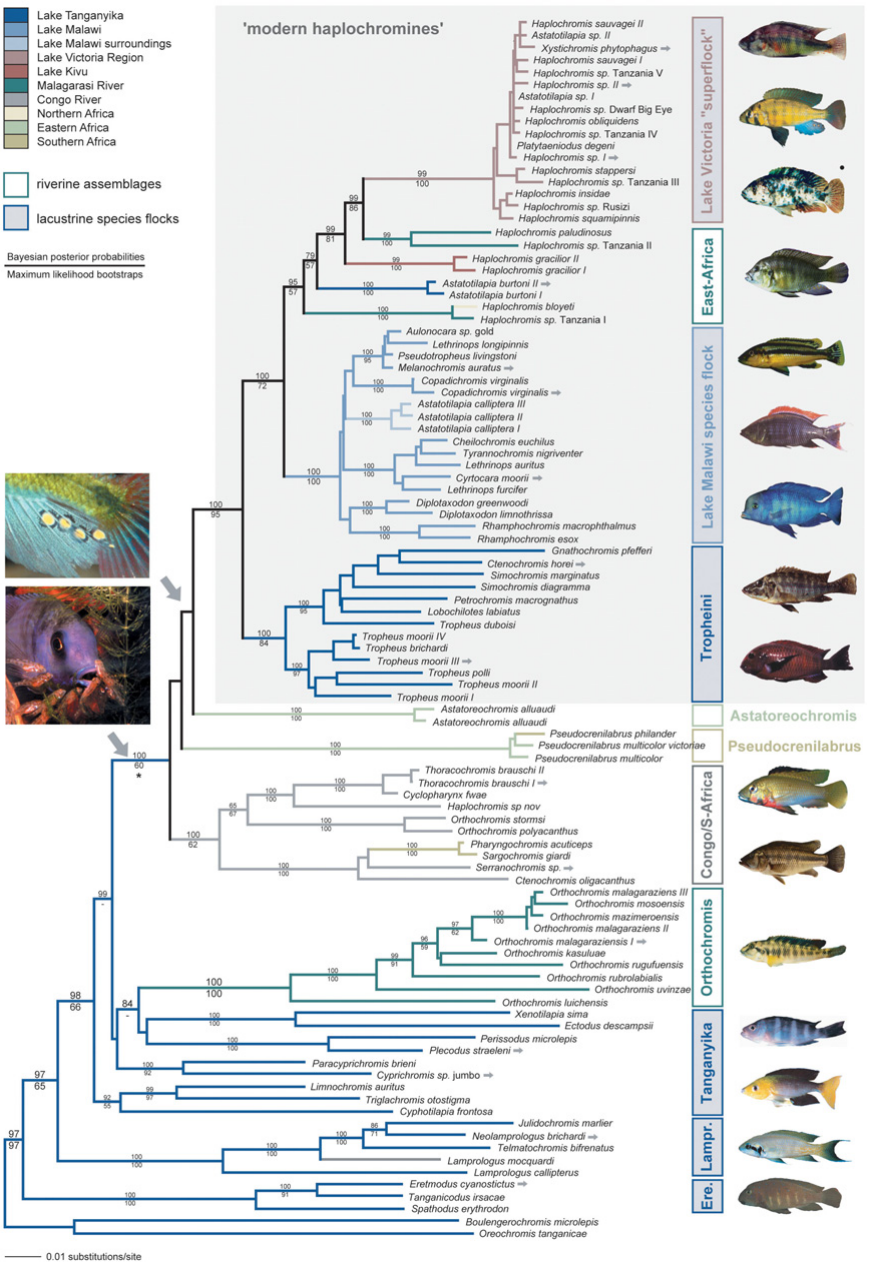
**Maximum likelihood phylogeny of the haplochromine cichlids **[general time-reversible model with gamma correction] based on 100 taxa. Numbers above the branches represent Bayesian posterior probabilities obtained with Mr. Bayes, numbers below the branches represent maximum-likelihood bootstraps (100 replicates, obtained with PAUP*). *Boulengerochromis microlepis *and *Oreochromis tanganicae*, two tilapiine cichlids in LT, were used as outgroup taxa [9, 15, 16, 63]. In accordance to previous studies [9, 15] we find that within the LT species flock the Eretmodini (Ere.) are placed as sister group to the Lamprologini (Lampr.) plus several LT tribes ("Tanganyika") including the *Orthochromis *assemblage from the Malagarasi plains, and the haplochromines *sensu lato*. The latter clade combines four distinct lineages, a Congolese/South-African- (CSA; ~150 species), the *Pseudocrenilabrus*- (3 species), the *Astatoreochromis*-lineage (3 species), and the modern haplochromines (~1,800 species). The modern haplochromines combine the LT Tropheini, the species flock of LM, several riverine lineages as well as the LV region superflock according to [4]. The haplochromines *sensu lato *are characterized by their breeding behavior; true egg-spots (*ocelli*) are likely to have evolved in the ancestor of the *Astatoreochromis*-lineage and the modern haplochromines. By contrast, the Malagarasi River *Orthochromis *are biparental caregivers [34] providing behavioral support for our molecular-based classification that excluded these fish from the haplochromines *sensu lato*. We note that several genera are polyphyletic and major taxonomic revisions will be required in the future to take our phylogenetic results into consideration. For example, *Orthochromis *of the Malagarasi River plains form a clade outside the remaining haplochromines in close affinity to the LT Ectodini (see also [9]) whereas *Orthochromis polyacanthus *and *O. stormsi*, which share derived features [12], fall – in accordance to their distribution – into the Congolese/South African clade. Other polyphyletic genera are *Astatotilapia*, *Ctenochromis*, and *Haplochromis*. The grey arrows next to some species names refer to the pictures on the right, the asterisk symbol marks the ancestor of the haplochromines *sensu lato*, the circle symbol marks a "piebald" ("orange blotched") form as found in the modern haplochromines only.

In the maximum likelihood tree of the dataset including 100 taxa (Fig. 3) the LT Eretmodini were placed as sister group to the LT Lamprologini, followed by a clade comprised by the two representatives of the Limnochromini (*Limnochromis auritus*, *Triglachromis otostigma*) plus *Cyphotilapia frontosa*, and all remaining taxa. Among these, a clade that includes four LT tribes (Cyprichromini, Ectodini, Limnochromini, Perissodini) plus the *Orthochromis *species from the Malagarasi drainage, which had so far been considered to belong to the Haplochromini, was recovered as sister group to the remaining haplochromine representatives. The latter, the haplochromines *sensu lato*, clustered into four distinct groups, with a Congolese/South-African lineage (CSA) sister to the *Pseudocrenilabrus*-, the *Astatoreochromis*-lineage, and the 'modern haplochromines' (*i.e. *a clade comprised by the LT Tropheini sister to the LM representatives, several East-African riverine lineages and the members of the LV region superflock). Bayesian inference revealed the same branching order for the different lineages. In the strict consensus topology of the 78,617 most parsimonious trees (unweighted tree length: 4400; tree not shown), the Eretmodini were again resolved as sister group to the Lamprologini, the Limnochromini plus *C. frontosa*, four LT tribes plus the Malagarasi *Orthochromis *and the haplochromines *sensu lato*. Here, the *Astatoreochromis*-lineage was resolved as most ancestral lineage, and as sister group to a clade comprised by the *Pseudocrenilabrus*- and the CSA lineage, and the modern haplochromines. Also, the neighbor-joining analysis recovered a tree (not shown) with the Eretmodini as sister-group to the Lamprologini, the Limnochromini plus *C. frontosa*, the four LT tribes including the Malagarasi River *Orthochromis *species, and the haplochromines *sensu lato*. In the neighbor-joining tree, the *Pseudocrenilabrus*-lineage occupied the most ancestral branch in the haplochromines *sensu lato*, followed by the Congolese/South-African clade, the *Astatoreochromis*-lineage and the modern haplochromines. A Shimodaira-Hasegawa test [19] revealed that there are no significant differences between the topologies obtained with the different algorithms (P < 0.05). Similarly, in the four-cluster likelihood mapping analysis [20] none of the three possible alternative branching orders among the four main lineages of the haplochromines *sensu lato *received support greater than 50%.

**Figure 4:**
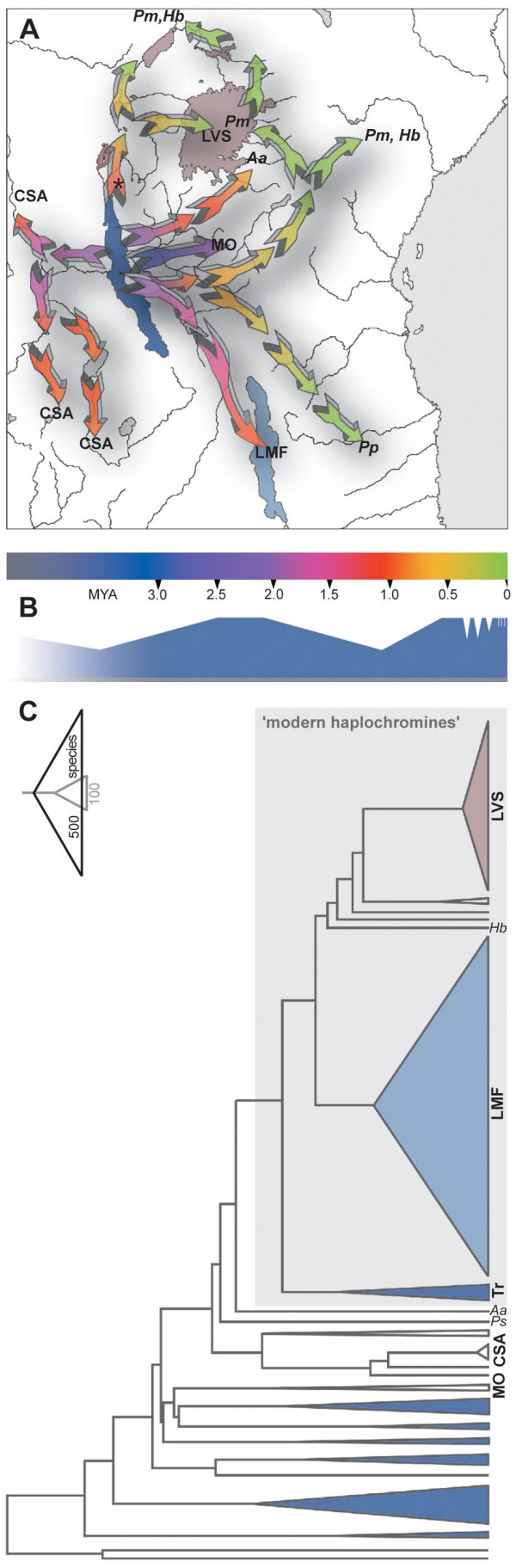
**The "out of Tanganyika" scenario of haplochromine evolution in Africa**. (*a*) Several haplochromine lineages independently left Lake Tanganyika and colonized large parts of Africa *via *past and present river connections. Some of these lineages seeded cichlid radiations in distant lakes. The phylogeographic scenario is in agreement with palaeo-geological reconstructions of the evolution of the East African Rift region. LT is the oldest of the rift lakes. Its central basin began to form between 9 and 12 MYA, the northern (8-7 MYA) and the southern basin (2–4 MYA) began to fill at later periods [76]; deepwater conditions exist since about 5–6 MYA [78]. LM (2–4 MYA) and LV (750,000 years) are considerably younger. (*b*) Proposed lake level of Lake Tanganyika during the last four million years [76, 77, 79] indicating major low- and high-stands. (*c*) Chronogram of the haplochromine evolution in Africa as reconstructed with r8s [72, 73] based on the maximum likelihood topology. The size of each clade represents its species number. The modern haplochromines are a recent and rapidly speciating lineage. Our molecular clock calibration suggested about 2 MYA (1.15 – 3.89 MYA) for the most recent common ancestor in the Congolese/Southern African lineage and ca. 2.4 MYA (1.22 – 4.02 MYA) for their split from the common ancestor with the *Pseudocrenilabrus*-, *Astatoreochromis*-, and modern haplochromine lineage. This lies in the range of the proposed high lake-level stand of LT between the minima at 3.5 MYA and 1.1 MYA (650–700 m below present level) [76, 77] making an overflow through the Lukuga valley possible, thus opening the connection between LT and the Congo drainage. The asterisks mark nodes with a significant burst of lineage diversification (*p*_*c *_< 0.01) [21]. Aa... *Astatoreochromis alluaudi*, CSA... Congolese/South African lineage, Hb... *Haplochromis bloyeti*, LMF... Lake Malawi species flock, LVS... Lake Victoria Region Superflock, MO... Malagarasi *Orthochromis *assemblage, Pm... *Pseudocrenilabrus multicolor*, Pp...*Pseudocrenilabrus philander*, Ps... *Pseudocrenilabrus-*lineage, Tr... Tropheini.

The dating of the major cladogenetic events (Fig. 4) found an age of 2.4 MYA (1.22 – 4.02 MYA) for the most recent common ancestor of the four lineages of haplochromines, and about 1.8 MYA (0.66 – 3.78 MYA) for the most recent common ancestor of the modern haplochromines. This latter value was smaller in all resampling replications pointing to a younger age of the modern haplochromines compared to the remaining three lineages of haplochromines *sensu lato*. The most recent common ancestor in the CSA-clade was estimated to have lived about 2.0 MYA (1.15 – 3.89 MYA), the split of the CSA lineage from the common ancestor with the *Pseudocrenilabrus*-, *Astatoreochromis*-, and modern haplochromine lineage was dated to about 2.4 MYA (1.22 MYA – 4.02 MYA). The test for shifts in the probabilities of speciation in the haplochromines *sensu lato *according to [21] suggested increased rates of lineage diversification (*p*_*c *_< 0.01) along three branches in the maximum likelihood, the Bayesian inference and the neighbor-joining trees: (i) the branch leading to *Astatoreochromis*-lineage plus the modern haplochromines; (ii) the branch leading to the modern haplochromines; and (iii) the branch leading to the LV region superflock (see Fig. 4c for the maximum likelihood tree). In the maximum parsimony strict consensus tree an increased rate of lineage diversification was found: (i) for the branch leading to the modern haplochromines; and (ii) for the branch leading to the LV region superflock.

**Figure 5:**
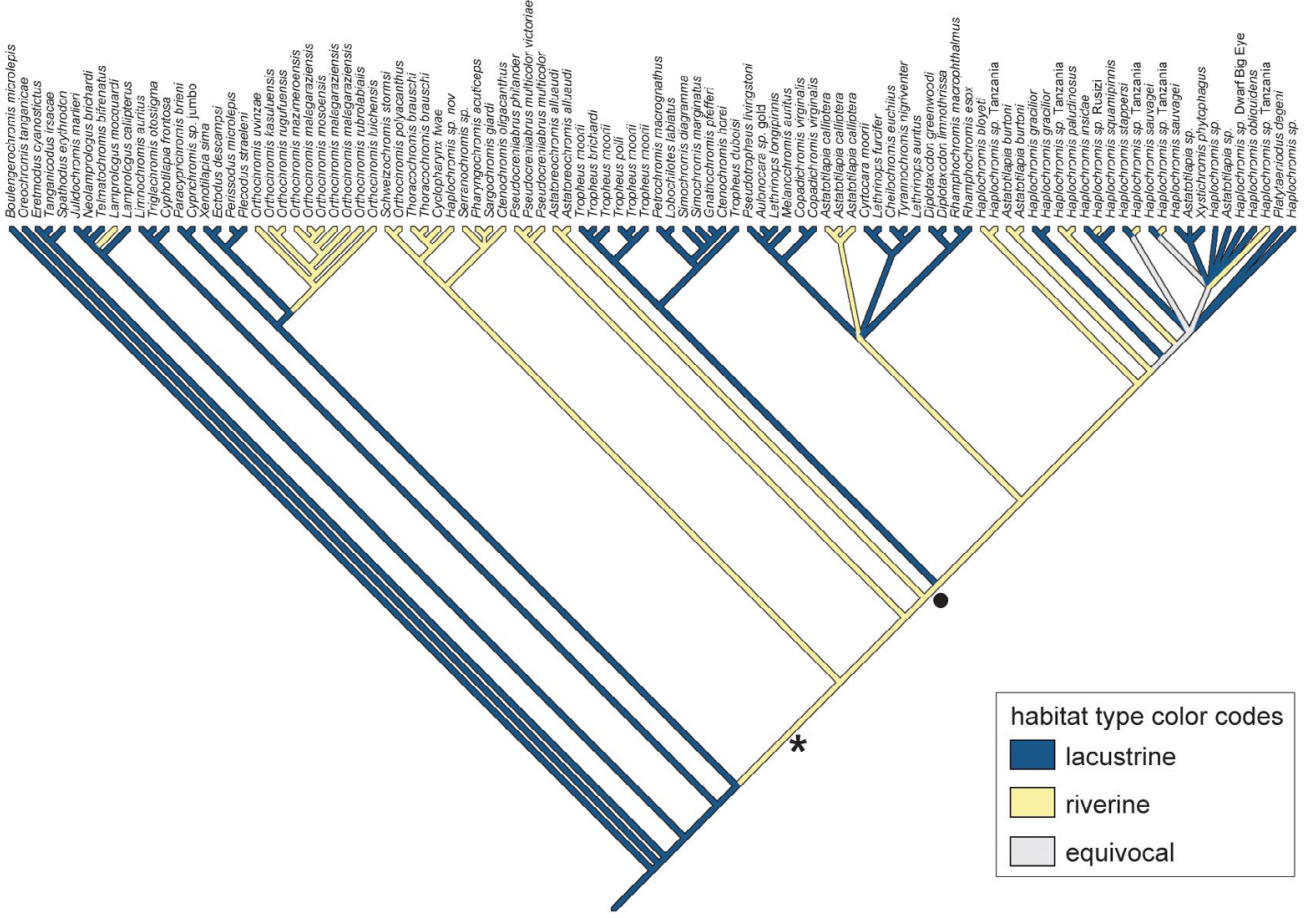
**Maximum parsimony reconstruction of habitat type **(lake *versus *river) using MacClade (the maximum likelihood reconstruction with Mesquite revealed analogous results) based on the maximum likelihood topology (see Fig. 3). The ancestor of the modern haplochromines (marked by a circle) is likely to have been a riverine species. This implies that also the Tanganyikan Tropheini originated from a riverine ancestor and re-colonized the lake where they presently form an abundant group in the rocky littoral zones.

The maximum parsimony and maximum likelihood character state reconstructions revealed that the characteristic maternal mouthbrooding behavior, where only the females incubate their fry in their buccal cavities, evolved in the common ancestor of the CSA lineage, the *Pseudocrenilabrus*-, the *Astatoreochromis*-lineage, and the modern haplochromines (see asterisk in Fig. 3). The true haplochromine-like egg-spots [10,22,23] are likely to have evolved in the common ancestor of the *Astatoreochromis*-lineage and the modern haplochromines (Fig. 3). Some members of the CSA lineage also show yellow or reddish markings on their anal fin. However, these markings do not represent real haplochromine-like egg-spots, which are characterized by an inner yellow or orange ring and an outer transparent and colorless ring [10,22,23]. Instead, the more homogenous markings seen in the CSA lineage might be viewed as an intermediate character state in the evolution of the species-specific egg-spots as found in the *Astatoreochromis*-lineage and in the modern haplochromines. The mapping of riverine *versus *lacustrine lifestyle onto the maximum likelihood topology suggested that the ancestor of the modern haplochromines was riverine (Fig. 5).

## Discussion

### Phylogeny and evolutionary origin of haplochromines

Mitochondrial DNA (mtDNA) sequence data have a long and successful history in the study of East African cichlid evolution (see *e.g., *[1,4,9,17,24,25]). Limitations with mtDNA have only been encountered when focusing on the phylogeny among extremely closely related species due to the possibility of the persistence of ancestral polymorphism (see *e.g., *[26]), or because of hybridization events (see *e.g., *[27-29]). Nevertheless, mtDNA sequences proved to be particularly suitable for the reconstruction of the East African cichlid phylogeny at the tribal level and for tribal assignments [9,15]; for phylogenetic reconstruction within older tribes [30,31]; and for phylogeographic analyses [4,32]. Also, because of the extremely fast rate of lineage formation in cichlids, nuclear and even some mitochondrial genes [1] are too slowly evolving to contain phylogenetic information (reviewed in [6]).

The different phylogenetic algorithms, with which we analyzed our data, revealed largely congruent results. In all analyses, and in agreement with previous results [9], we found that the Eretmodini are placed as sister group to the substrate spawning Lamprologini – with an estimated number of up to 100 species the most species-rich tribe in LT – plus several LT tribes and all haplochromine representatives (see Fig. 3 for the maximum likelihood tree). The molecular phylogenies thus corroborate that all haplochromines are ultimately derived from LT cichlids and that their ancestor(s) are likely to have left LT secondarily.

We consider all species that belong to the monophyletic group descending from this ancestor (asterisk in Fig. 3), to being haplochromines *sensu lato*. These are further divided into four distinct groups, a Congolese/Southern African lineage (CSA), the genera *Pseudocrenilabrus *(Ps.) and *Astatoreochromis *(As.), and the modern haplochromines (MH). While the respective monophyly of these four lineages was supported by high bootstrap values and Bayesian posterior probabilities, our analyses could not unambiguously resolve the exact relationships between these four lineages [maximum likelihood and Bayesian inference (see Fig. 3): (CSA, (Ps., (As., (MH)))); maximum parsimony: (As., ((Ps., CSA), (MH))); neighbor joining: (Ps., (CSA, (As., (MH))))]. An evaluation of these alternative hypotheses by means of a Shimodaira-Hasegawa test [19] and a four-cluster likelihood mapping analysis [20] indicated that none of these alternative branching orders receives significantly more support than the others. This suggests that the four lineages of the haplochromines *sensu lato *evolved almost contemporaneously from a common ancestor. This is further supported by the observation of relatively short branches interrelating these four lineages and the generally low bootstrap values and Bayesian posterior probabilities supporting the corresponding relationships proposed by the different algorithms.

The CSA lineage is composed of several widespread and moderately species-rich groups of the Congo drainage and Southern Africa and consists of two main clades: A clade with a species from the Congo drainage (*Ctenochromis oligacanthus*) ancestral to Southern African genera (*Serranochromis*, *Sargochromis*, *Pharyngochromis*) was resolved as sister group to a clade comprised by solely Congo drainage taxa (*Orthochromis*, *Haplochromis sp. nov., Cyclopharynx *and *Thoracochromis*). Within the *Pseudocrenilabrus*- and *Astatoreochromis*-clades, branch lengths were relatively short and a more detailed phylogeographic sampling would be necessary to resolve the relationships between the different geographic lineages.

The modern haplochromines consist of species flocks of an unparalleled diversity. They include the endemic LT tribe Tropheini (~25 species) sister-group to a clade comprised by the entire species flock of LM (~1,000 species) and several East African riverine and lacustrine lineages (~200 species) plus the LV region superflock (~600 species). With approximately 1,800 species this – here phylogenetically defined – monophyletic lineage makes up about 7% of all known teleost fish.

### *Astatotilapia*, *Thorachochromis*, and *Orthochromis *are polyphyletic genera

Our phylogenies show that several genera are in fact polyphyletic, and major taxonomic revisions will be required in the future to take our phylogenetic results into consideration. For example, and in agreement with previous studies (see *e.g.*, [4,14,33]), *Astatotilapia *emerges as polyphyletic genus, with representatives assigned to both the East African riverine clade in the modern haplochromines and the LV region superflock.

The genus *Thoracochromis*, represented in our analysis by *T. brauschi*, has also been shown to be polyphyletic before [14], with *T. brauschi *from the Congo drainage as a more ancestral lineage, and *T. petronius *and *T. pharyngealis *from the Nile drainage with affinities to the LV region superflock (note that the Nile River *Thoracochromis *of [14] are consequently listed as *Haplochromis *in [34]). The placement of *T. brauschi *as sistergroup to *T. petronius *and *T. pharyngealis *plus the remaining representatives of the LV region superflock in the AFLP based phylogeny of [14] seems to contradict our mtDNA based results in which *T. brauschi *was identified as member of the CSA lineage. However, the reported branching order did not receive considerable bootstrap support in [14]. Also, the choice of *Astatoreochromis alluaudi *(mislabeled as *Astatotilapia alluaudi *in [14]) as single outgroup species seems problematic, as our present analyses (see above) and former results [4] indicate that *A. alluaudi *and not *T. brauschi *is more closely related to the modern haplochromines (and, thus, also to the LV region superflock). Further analyses including nuclear DNA sequence data and more taxa assigned to the genus will be necessary to address this problem.

Based on the phylogeny it is apparent that the *Orthochromis *lineage, which is confined to the Malagarasi River system and two isolated rivers, East of LT [35], is not part of the radiation of haplochromine cichlids. This is further supported by the breeding behavior of these fish: While the Malagarasi River *Orthochromis *are biparental caregivers [35], the haplochromines *sensu lato *are all maternal mouthbrooders. Thus, the *Orthochromis*-species from the Malagarasi area, a group of exclusively riverine fish, should be placed into its own tribe. The name *Orthochromis *is, however, also used for riverine species from the Congo drainage. In our analyses, *Orthochromis polyacanthus *and *O. stormsi *from the Congo River system fall – according to their distribution – into the CSA-clade leaving also the genus *Orthochromis *polyphyletic (see also [9]). We suggest to using *Orthochromis *for the CSA lineage representatives, since *O. polyacanthus *was the first species of the genus to be described [36], and Schwetzochromini (as tribe name) and *Schwetzochromis *(as genus name) for the Malagarasi area species, since this name was repeatedly used for some species of that complex (see *e.g.*, [9,34,35]).

### Phylogeography and phylochronology

We note that the application of a molecular clock for estimating divergence times has the potential of not being without problems for several reasons (see *e.g.*, [37,38]). However, a molecular-clock-based time estimate does surely provide an approximate framework for phylogeographic inferences. Our phylogeographic scenario (Fig. 4a), which is derived from the maximum likelihood phylogenetic and molecular clock analyses, suggests that several lineages independently left LT to colonize surrounding river systems and consequently other lakes in East Africa. The molecular clock calibration based on the chronogram generated with r8s [39] (Fig. 4b,c) yielded about 2.4 MYA (1.22 – 4.02 MYA) for the split of the CSA lineage from the common ancestor of the haplochromines *sensu lato*, and about 2 MYA (1.15 – 3.89 MYA) for the first branching events within the CSA clade. The spread of Congo drainage taxa into southern river systems occurred at a later stage, most likely at the relatively shallow watershed between upper branches of the Congo River and the Zambezi River – a scenario that is also supported by the placement of *Serranochromis sp. *(from Lake Mweru in the upper Congo) as sister group to the Zambezi/Southern African genera *Sargochromis *and *Pharyngochromis *in our phylogenies. However, further sampling in that area would be necessary to reconstruct the southward spread of the CSA lineage.

At essentially the same time as the CSA lineage, the ancestors of the *Pseudocrenilabrus- *and the *Astatoreochromis-*lineage diverged from their common ancestor. Despite their large distributional ranges – they also colonized the LV (both lineages) and LM (*Pseudocrenilabrus*) region – the genera *Astatoreochromis *and *Pseudocrenilabrus *never underwent considerable speciation. The three described *Astatoreochromis *species occur in the LV region including Lakes Edward and George (*A. alluaudi*), in the rivers Rusizi and Lukuga (*A. straeleni*), and in the Malagarasi River (*A. vanderhorsti*). The three species of *Pseudocrenilabrus *occur from the Nile system to the LV region (*P. multicolor*), in Eastern- and Southern Africa including LM (*P. philander*), and in the central Congo basin (*P. nicholsi*). All analyzed representatives are relatively closely related suggesting a recent spread of these lineages in East Africa. However, we did not include *P. nicholsi*, which is morphologically different from *P. multicolor *and *P. philander *and would – if it really belonged to the *Pseudocrenilabrus*-lineage – represent the only haplochromine in the Congo drainage that is not a member of the CSA lineage.

The most recent common ancestor of the modern haplochromines was dated to have existed about 1.8 MYA (0.66 – 3.78 MYA) (Fig. 4). This ancestral lineage forms the crucial phylogenetic and biogeographic link between the species flocks of all three East African Great Lakes, and its discovery documents the existence of much earlier hypothesized fish-accessible waterways between these waterbodies [40,41]. Apparently, the Malagarasi River (and possibly the Rusizi) played a major role for the dispersal of these fishes, since many modern haplochromine lineages occur in these drainages and in lakes South-Eastern and North of LT exclusively, which argues against the view that LM haplochromines originated from Zambezi River stocks [41]. Whether or not Lake Rukwa has ever acted as link between the faunas of LT and LM [41] cannot be answered by our data. Lake Rukwa seems to have overflowed at its maximum levels into LT several times. However, Lake Rukwa has also become very shallow in recent geological times and it might have dried up completely [41] eradicating its original fauna. At present, Lake Rukwa harbors haplochromines that belong to the East-African riverine clade in Figs. 2, 3[4,25].

Our analyses also recovered another closely related lineage to the LV region superflock in the East-African riverine clade, in addition to *Haplochromis gracilior *form Lake Kivu [4]. This lineage includes *H. paludinosus *that occurs in the Malagarasi (which was already suggested by [33]), as well as undescribed species from Tanzania and Lake Edward (Figs. 2, 3). It is, however, unclear by which waterway haplochromine cichlids once colonized Lake Kivu. The flow of the Rusizi, presently from Lake Kivu into LT with the Panzi falls as strong barrier for fish migration, might actually have been reversed before the uplift of the Virunga volcanoes north of Lake Kivu as suggested by deposits of fossil LT mollusks and fluviatile sands in the upper Rusizi valley [41,42]. This connection could possibly explain how haplochromines of LT origin were able to colonize Lake Kivu about 1.5 million years ago (Fig. 4).

### Evolutionary key-innovations of haplochromines

One of only few synapomorphies of the haplochromines *sensu lato *is the particular type of cranial apophysis for the upper pharyngeal bones [12]. The distinctive organization of the pharyngeal apophysis, a second set of jaws that is functionally decoupled from the oral ones [43], is characteristic to all cichlids and has been interpreted as prominent feature that – because of its adaptability – contributes to the cichlids' evolutionary success [2,3,10,43]. It is, however, not evident how the relatively minor morphological modification of part of that structure in the haplochromines [12] might function as an evolutionary key-innovation. Interestingly, however, all haplochromines *sensu lato *are maternal mouthbrooders with the females alone incubating the eggs in their buccal cavities [10,12]. Mouthbrooding, which is regarded as an adaptation to predation pressure [44-46], has evolved several times independently and in diverse behavioral modes in cichlids [10,22,47,48]. The characteristic *maternal *mouthbrooding behavior displayed by haplochromines is believed to being a derived character state [35,46,47]. Mouthbrooding strongly limits the number of eggs and fry that can be raised and might have led to generally much smaller population sizes, which has, for example, population genetic implications on fixation of alleles, and might result in smaller effective population sizes. Furthermore, mouthbrooding species may be considered to being promising colonizers of new habitats, since only a single mouthbrooding female is necessary for the founding of a new population.

An eminent feature of several female mouthbrooding cichlid genera is the occurrence of egg-spots on the anal fins of males. In some species also females show such ovoid markings, but these are smaller and much less conspicuous than in males. Also, some species of the modern haplochromines, *e.g.*, some deep-water lineages of LM, have lost their egg-spots secondarily. In mimicking real eggs to attract females, these egg-spots function as natural releasers [22,23], or intra-specific sexual advertisement [44], apparently serving to ensure a greater fertilization success of the eggs by bringing about greater proximity of the female's mouth to the male's genital opening. Based on the molecular phylogeny, we could trace the origin of the characteristic egg-spots (*ocelli*) [10,23] to the common ancestor of the *Astatoreochromis*-lineage and the modern haplochromines. There are other cichlid species in which males display yellow or red marks on their pelvic, dorsal or anal fins, but only in these lineages true egg-spots on the males' anal fins with a yellow, orange or red center and a colorless/transparent outer ring [10,22] are found. Interestingly, the branch leading to the *Astatoreochromis*-lineage and the modern haplochromines is the one with a pronounced potential for an increased rate of speciation (see Fig. 4c). Based on the character state reconstructions (Fig. 5) it seems likely that this ancestor was riverine. Thus, it may be concluded that the egg-spots first evolved in a haplochromine cichlid that inhabited a turbid riverine environment, where these conspicuous markings would seem to be particularly effective and necessary for intra-specific communication.

Another innovation that further distinguishes the exceptionally species-rich modern haplochromines from all other cichlids is the occurrence of numerous color morphs, often accompanied by sexual color dimorphism. Inter- and intra-specific polychromatism combined with maternal mouthbrooding involving egg-spots as releasers can be hypothesized to being permissive for sexual selection through female choice and, hence, the haplochromines' propensity for species formation, as sexual selection is probably a major causal factor in the origin of isolating mechanisms and the maintenance of reproductive isolation [18,49-53]. These distinctive features of the modern haplochromines, that have arisen just in their ancestor, in combination with the numerous ecological niches that are provided by the large East African lakes might thus have induced a considerable increase of the haplochromines' evolutionary potential. The importance of large waterbodies for the evolution of the modern haplochromines is reflected by the fact that these cichlids only radiated in lakes (and species number rather correlates to the size, but not to the age, of a lake), whereas the riverine lineages are all species-poor albeit often widespread (Figs. 1, 3).

### Replicate adaptive radiations of the 'modern haplochromines'

A common feature of many adaptive radiations is that their founders are believed to have had a more generalist's lifestyle, while adaptive radiations themselves are defined by being composed of highly specialized species with narrower niche widths [54,55]. Theory predicts that generalists more likely have better dispersal abilities and are expected to be able to adapt readily to novel environmental settings [55]. A generalist ancestor scenario fits well with the diversification of haplochromine cichlids. Generalist riverine species of the genera *Astatoreochromis*, *Astatotilapia*, *Pseudocrenilabrus*, and *Haplochromis *(*e.g.*, *bloyeti*), are ancestral to the adaptive radiations of the Tropheini of LT, and/or the radiations of LM and the LV region superflock. These genera are widely distributed and not confined to Eastern Africa, and they are the only ones that could inhabit the waterways that – over geological time spans – connected the lakes of Eastern Africa.

The phylogeny presented here (Fig. 3) reveals that modern haplochromines gave rise to several major adaptive radiations; the most prominent ones are those of LM and LV. Interestingly, it uncovers that also the radiation of the Tropheini from LT [30] must now be considered as an additional radiation of the modern haplochromines, corroborating the much older perception that LT accommodates several independent species flocks [56]. It is further suggested by mapping the fishes' lifestyle onto our molecular phylogeny that the highly specialized Tropheini are descendents of a river-living species. This implies that the ancestor of the Tropheini successfully re-entered the lake habitat and evolved into the presently dominant group in the rocky littoral zone of LT. Thus, this lineage of modern haplochromines managed to occupy "empty niches" in an apparently "full" ecosystem, as all remaining tribes, which now account for about 200 species, had already been established when the ancestor of the Tropheini secondarily entered LT (Figs. 2, 3). The observation that these fish underwent an independent adaptive radiation in LT underlines the haplochromines' propensity for speciation.

In an apparent contrast to most other known examples of adaptive radiations [55] is the finding that the generalist ancestors of the haplochromine species flocks were derived from already highly diverse and specialized LT endemics (Fig. 3). Therefore, specialization may not be an "evolutionary one-way street", but rather some lineages have reversed their level of specialization, *i.e.*, generalists arose from highly specialized lineages, yet, apparently retained their high propensity for speciation and level of evolvability (see [57]). The faunal revolution of LT's radiation of cichlids was thus not confined to the lake habitat itself (see also [8,9,58]), but it effectively involved large parts of Africa via the intermediate step of repeatedly evolving generalist riverine lineages – in much the same way as the adaptive radiation in LV produced haplochromine species that secondarily colonized surrounding rivers [4].

Our phylogeny of haplochromines provides strong support for replicate adaptive radiations in East African cichlids. The concept of replicate radiations, in which the same sequence of adaptations to ecological niches evolved repeatedly in lineages that inhabit similar environments, has been developed based on sympatric species pairs of fishes in postglacial lakes and on the *Anolis *lizard ecomorphs on different islands of the Greater Antilles [59-61]. Our inclusive phylogenetic and phylogeographic study shows that similar ecological types of cichlids in the different East African lakes evolved independently (see also [2,17]), yet it also shows that the convergent ecotypes in the species flocks of LM, LV, Lake Kivu as well as in the Tropheini [2,10,12,13,17] arose from the same ancestral phenotype in the ancestor of the modern haplochromines. We suggest that a combination of behavioral (maternal mouthbrooding) and morphological innovations (egg-spots, color polymorphisms, pronounced sexual dichromatism) as well as ecological opportunities (after the colonization of large lakes) might have predestined this particular lineage to give rise to these replicate adaptive radiations.

It has been noted before that lineages of LT origin have left the lake secondarily (there are, for example, about five lamprologine species that are found in the Congo and Malagarasi Rivers) [9,58]. Here, we show that the entire haplochromine diversity has its origin in LT corroborating the view that ancient lakes not only preserve biodiversity but also act as biodiversity hotspots, genetic reservoirs and cradles from which new lineages evolve [4,8,9]. What remains to be answered is where the LT cichlids originated and to what extent a proposed and meanwhile desiccated Pliocene lake in the Congo plains [41,42] was the source of the ancient LT lineages, pushing back even further the onset of replicate adaptive radiations in East African cichlids.

## Conclusion

Our phylogenetic analyses that include representatives of all major haplochromine lineages show that all haplochromines are derived from Lake Tanganyika cichlids. While the *Orthochromis *species of the Malagarasi area apparently do not belong to the radiation of the haplochromine cichlids and should be placed into a new tribe, we defined four new lineages within the haplochromines *sensu lato*: A clade combining Congolese and South-African genera (CSA-lineage), the *Pseudocrenilabrus*-, the *Astatoreochromis*-lineage, and the exceptionally species-rich modern haplochromines. The ca. 1,800 species of modern haplochromines are comprised of the entire haplochromine species flocks of Lake Malawi and the Lake Victoria region, some 200 riverine and lacustrine species, as well as the Tanganyikan Tropheini, which are likely to have evolved from a riverine ancestor and secondarily colonized Lake Tanganyika. This proposed "out of Tanganyika" scenario of haplochromine evolution is in agreement with the geological and palaeo-geological history of East Africa. Based on a character-state reconstruction from this new phylogeny, we were able to discover the evolution of several key-innovations that arose in the lineage leading to the modern haplochromines. These character reconstructions suggest that a combination of behavioral (maternal mouthbrooding) and morphological characteristics (egg-spots, color polymorphisms, pronounced sexual dichromatism) as well as ecological opportunities (after the colonization of large lakes) might have predestined this particular lineage to give rise to replicate adaptive radiations and, therefore, be causally related to the extraordinary success of these particular cichlid fish radiations.

## Methods

### Specimen information and DNA methods

For this study, a total of 304 specimens were analyzed. We combined all available GenBank entries from previous studies [4,9,15,25,62] with 180 newly determined DNA sequences. Table 1 [see Additional file 1] lists specimen information, geographic origin of the specimens, names of collectors, and GenBank accession numbers. When available, voucher specimens have been deposited at the Royal Museum for Central Africa, Tervuren, Belgium.

Sample preparation, polymerase chain reaction (PCR) amplification and DNA sequencing have been performed as described elsewhere [9] for both mitochondrial DNA segments the complete non-coding control region and the entire NADH Dehydrogenase Subunit II (ND2) gene. Forward and reverse sequences have been assembled using the computer programs Sequence Navigator (Applied Biosystems, USA) and Sequencher (GenCodes, USA).

### Phylogenetic reconstruction and hypotheses testing

The complete sequences of the mitochondrial control region (895 bp) and the ND2 gene (1,047 bp) were aligned using the computer program Clustal W [63]; alignments have been further adjusted by eye. Up to 34 gaps had to be included in the control region alignment, which were coded as indels. Due to missing data on the 5'-end of the control region in about one quarter of the taxa, a terminal section of 34 bp has been excluded from the phylogenetic analysis, leading to an alignment of 1,908 bp for the combined dataset.

For phylogenetic reconstruction we performed maximum likelihood, maximum parsimony and neighbor-joining methods in parallel using the computer program PAUP* 4.0b10 [64]. Two taxa belonging to tilapiine cichlids were included as outgroup based on previous phylogenetic analyses using mitochondrial DNA [9,15,16], nuclear DNA [65] and SINE insertion patterns [66,67] demonstrating that the Tilapia-lineages are ancestral to all remaining LT tribes (but excluding *Tylochromis*, which is ancestral to the Tilapiini). We did not include representatives of three LT tribes – the Bathybatini, Trematocarini and Tylochromini -, as these ancient lineages were shown to have existed before the primary radiation of mouthbrooders in LT [9,15,67]. After an initial neighbor-joining analysis including the control region sequences of all 304 specimens, we reduced the dataset to 100 taxa based on the obtained topology. This reduction was necessary to allow computational feasibility for maximum likelihood and maximum parsimony analyses. The optimal model of molecular evolution for the maximum likelihood analysis was determined in a likelihood-ratio test running the computer program Modeltest v3.06 [68]. For the heuristic maximum likelihood search of the combined dataset we used the general-time-reversible model of molecular evolution, with a gamma shape parameter of 0.7937 and a proportion of invariable sites of 0.3426. Due to the many closely related taxa in the dataset, maximum parsimony analyses were completed for 10^10 ^rearrangements. For neighbor joining, we used the HKY model and conducted a bootstrap analysis with 5,000 replicates. Bootstrap analyses for maximum likelihood were performed with 100 replicates and for maximum parsimony with 1,000 replicates. We also applied Bayesian inference of phylogeny with Mr. Bayes 3.0b4 [69] running four Metropolis Coupled Monte-Carlo-Markov-Chains in parallel for 250.000 generations, using the general-time-reversible model with gamma correction (six types of substitutions), and excluding 5 % of the trees as burn-in. The obtained topologies from the different phylogenetic algorithms were evaluated by means of a nonparametric Shimodaira-Hasegawa test [19] under a resampling-estimated log-likelihood as implemented in PAUP* [64]. To estimate the support for distinct internal branches critical for our interpretations, we performed a four-cluster likelihood mapping analysis with the program PUZZLE 5.0 [20,70] in which we grouped the taxa of the haplochromines *sensu lato *into four groups according to the four lineages that were recovered from the phylogenetic analyses (CSA-, *Pseudocrenilabrus*-, *Astatoreochromis*-lineage, and modern haplochromines).

To test whether or not shifts in the probabilities of speciation occurred on certain branches of the obtained phylogeny, we quantitatively tested the fit of this tree to a Markovian null model in which the probability of speciation is equally distributed along branches. Therefore, we calculated the cumulative probability *p*_*c *_[21] for relevant branches based on the different trees. We then mapped lifestyles (riverine *versus *lacustrine), breeding behavior, and the occurrence of true egg-spots on the maximum likelihood tree using MacClade 4.0 (Sinauer, Sunderland, MA) for maximum parsimony character state reconstructions and Mesquite [71] for maximum likelihood reconstructions. To tentatively date the major cladogenetic events in the haplochromines, we constructed a chronogram based on a maximum likelihood tree with constraint molecular clock (see above for search parameters). Therefore, we used the computer program r8s [72] applying the local molecular clock method and an optimization via the truncated newton method [73]. Confidence intervals were assessed by means of a bootstrap approach. We simulated 25 bootstrap matrices with Mesquite and, for each matrix, constructed a maximum likelihood tree (general-time-reversible model; model parameters, gamma shape correction and proportion of invariable sites being estimated from each matrix; rearrangements limited to 1,000). The resulting trees were then analyzed with r8s as described above, and in addition with the minimum and maximum values of geological datings separately, in order to define upper and lower bounds. As calibration points, we used an age estimate for the LM species flock of about one million years [74,75], the maximum age of 200.000 years for the LV region superflock [1,4,25] as well as the time window for the Lukuga connection between LT and the Congo system (between the minima at 3.5 MYA and 1.1 MYA) [76,77]. An escape of LT lineages into river systems draining into the lake was possible at any time, whereas the only connection to the Congo system, the Lukuga, was available at periods of high lake level stands only. The Lukuga channel, the only outlet of LT, was dry when first seen in 1874 by Cameron, but four years later the lake overflowed. Since then, the Lukuga was repeatedly flooded. Intermittent connections with the Congo River system in the late Pliocene/early Pleistocene were suggested on the basis of large lake level fluctuations in LT during periods with increased precipitation [41,76-79]. The resulting molecular clock rate fits well with previously used rates in East African cichlids [4,75].

## List of abbreviations used

As., *Astatoreochromis*; bp, base pairs; CSA, Congo/South Africa; LM, Lake Malawi; LT, Lake Tanganyika; LV, Lake Victoria; MH, modern haplochromines; mtDNA, mitochondrial DNA; MYA, million years ago; ND2, NADH Dehydrogenase Subunit II; Ps., *Pseudocrenilabrus*.

## Authors' contributions

WS, EV, and AM designed the study and were involved in sampling. WS and TM carried out the molecular work and the analyses. All authors contributed to the preparation of the manuscript. They read and approved the final version.

## Supplementary Material

Additional File 1 **Table 1 – Specimen information, geographic origin and GenBank accession numbers of all taxa included in this study**. This table lists the species and tribe names, geographic origin, source of specimens, names of collectors and collection numbers (if available), and GenBank accession numbers for both mitochondrial gene segments. The taxa that are included in Fig. 3 are marked by a circle, specific taxon labels in Fig. 3 are depicted in the "Label" column. The clade names according to Figs. 2 and 3 are shown in the last column. Tribe names are according to [7]. Click here for file
