# Supplementary material for: Out of Tanganyika: Genesis, explosive speciation, key-innovations and phylogeography of the haplochromine cichlid fishes
Source: BMC Evol Biol. 2005 Feb 21;5:17. doi: 10.1186/1471-2148-5-17 (PMC554777; doi:10.1186/1471-2148-5-17)
Supplement: Additional File 1 — Table 1 – Specimen information, geographic origin and GenBank accession numbers of all taxa included in this study. This table lists the species and tribe names, geographic origin, source of specimens, names of collectors and collection numbers (if available), and GenBank accession numbers for both mitochondrial gene segments. The taxa that are included in Fig. 3 are marked by a circle, specific taxon labels in Fig. 3 are depicted in the "Label" column. The clade names according to Figs. 2 and 3 are shown in the last column. Tribe names are according to [7]. [file 1471-2148-5-17-S1.pdf]

Table 1. Specimen information, geographic origin and GenBank accession numbers of all taxa included in this study. This table lists the species and tribe names, geographic origin, source of specimens, names of collectors and collection numbers (if available), and GenBank accession numbers for both mitochondrial gene segments. The taxa that are included in Fig. 3 are marked by a circle, specific taxon labels in Fig. 3 are depicted in the "Label" column. The clade names according to Figs. 2 and 3 are shown in the last column. Tribe names are according to Poll [7].

abbreviations: Aa… Astatoreochromis alluaudi lineage, CSA… Congolese/South African lineage, LMF… Lake Malawi species flock, LVS… Lake Victoria Region Superflock, MO… Malagarasi Orthochromis assemblage, EAR… East African riverine cichlids, Ps... Pseudocrenilabrus lineage, Tr... Tropheini, LT… Lake Tanganyika species flock

* * * * * * Taxonomy * * * * * *		* * * * * * * * * * * * * Sample Information * * * * * * * * * * * * *			* GenBank accession number				
**Tribe**	**Taxon**	**Origin**	**Source**	**Collector (Collection Number)**	**control region**	**ND2**	**Fig. 2**	**Label**	**Clade**
Cyprichromini	*Cyprichromis sp. "jumbo"*	Lake Tanganyika	Salzburger et al. 2002 [9]	n/a	AF400699	AF398224	•		LT
	Paracyprichromis brieni	Lake Tanganyika	Salzburger et al. 2002 [9]	n/a	AF400700	AF398223	•		LT
Ectodini	*Ectodus descampsii*	Lake Tanganyika	Salzburger et al. 2002 [9]/Koblmüller et al. 2004 [35]	n/a	AF400703	AY337790	•		LT
	Xenotilapia sima	Lake Tanganyika	Salzburger et al. 2002 [9]/Kocher et al. 1995 [15]	n/a	AF400706	U07270	•		LT
Eretmodini	*Eretmodus cyanostictus*	Lake Tanganyika	Salzburger et al. 2002 [9]	n/a	AF400707	AF398220	•		LT
	Spathodus erythrodon	Lake Tanganyika	Salzburger et al. 2002 [9]	n/a	AF400708	AF398218	•		LT
	Tanganicodus irsacae	Lake Tanganyika	Salzburger et al. 2002 [9]	n/a	AF400709	AF398219	•		LT
Haplochromini	*Astatoreochromis alluaudi*	Lake Victoria Region (L. Kanyaboli)	this study	R. Abila (R281-2002)	AY929969	AY930075	•	II	Aa
	Astatoreochromis alluaudi	Lake Victoria Region	Nagl et al. 2000 [25]	n/a	AF213618	-			Aa
	Astatoreochromis alluaudi	Lake Victoria Region (L. Kanyaboli)	this study	R. Abila (R575-2002)	AY929997	-			Aa
	Astatoreochromis alluaudi	Lake Victoria Region (L. Kanyaboli)	this study	R. Abila (R192-2002)	AY929996	-			Aa
	Astatoreochromis alluaudi	Lake Victoria Region (L. Kanyaboli)	this study	R. Abila (R101-2002)	AY929966	AY930071	•	I	Aa
	Astatoreochromis alluaudi	Lake Victoria Region (L. Kanyaboli)	this study	R. Abila (R044-2002)	AY929995	-			Aa
	Astatoreochromis alluaudi	Lake Victoria Region (L. Kanyaboli)	this study	R. Abila (R576-2002)	AY929998	-			Aa
	Astatoreochromis alluaudi	Lake Victoria Region	Nagl et al. 2000 [25]	n/a	AF213616	-			Aa
	Astatotilapia burtoni	Lake Tanganyika	Salzburger et al. 2002 [9]/Klett & Meyer 2002 [16]	n/a	AF400710	AF317266	•	I	EAR
	Astatotilapia burtoni	Lake Tanganyika	this study	aquarium, T62	AY930000	-			EAR
	Astatotilapia burtoni	Lake Tanganyika	this study	aquarium, T63	AY930001	-			EAR
	Astatotilapia burtoni	Lake Tanganyika	this study	aquarium, H19	AY929999	-			EAR
	Astatotilapia burtoni	Lake Tanganyika	this study	L. De Vos (31-02/6/92). T34	AY929955	AY930060	•	II	EAR
	Astatotilapia calliptera	Lake Malawi	this study	I. Kornfield (A22)	AY929977	AY930090	•	III	LMF
	Astatotilapia calliptera	Lake Malawi	Shaw et al. 2000 [62]	n/a	AF298938	AF305277	•	II	LMF
	Astatotilapia calliptera	Lake Malawi	Shaw et al. 2000 [62]	n/a	AF298940	AF305279	•	I	LMF
	Astatotilapia calliptera	Lake Malawi	Shaw et al. 2000 [62]	n/a	AF298941	-			LMF
	Astatotilapia nubila	Lake Victoria	Nagl et al. 2000 [25]	n/a	AF213524	-			LVS
	Astatotilapia nubila	Lake Victoria	Nagl et al. 2000 [25]	n/a	AF213536	-			LVS
	Astatotilapia sp.	Lake Victoria Region (L. Kanyaboli)	this study	R. Abila (R184-2002)	AY929967	AY930072	•	II	LVS
	Astatotilapia sp.	Lake Victoria Region (L. Kanyaboli)	this study	R. Abila (R185-2002)	AY930045	AY930073	•	I	LVS
	Astatotilapia sp.	Lake Victoria Region (L. Kanyaboli)	this study	R. Abila (R183-2002)	AY930003	-			LVS
	Astatotilapia sp.	Lake Victoria Region (L. Kanyaboli)	this study	R. Abila (R182-2002)	AY930002	-			LVS
	Astatotilapia velifer	Lake Victoria	Nagl et al. 2000 [25]	n/a	AF213550	-			LVS
	Astatotilapia velifer	Lake Victoria	Nagl et al. 2000 [25]	n/a	AF213551	-			LVS
	Astatotilapia velifer	Lake Victoria	Nagl et al. 2000 [25]	n/a	AF213552	-			LVS
	Astatotilapia velifer	Lake Victoria	Nagl et al. 2000 [25]	n/a	AF213553	-			LVS
	Aulonocara sp "gold"	Lake Malawi	Shaw et al. 2000 [62]	n/a	AF398947	AF305286	•		LMF
	Cheilochromis euchilus	Lake Malawi	this study	I. Kornfield	AY929979	AY930092	•		LMF
	Copadichromis virginalis	Lake Malawi	Shaw et al. 2000 [62]	n/a	AF298944	AF305283	•		LMF
	Copadichromis virginalis	Lake Malawi	Shaw et al. 2000 [62]	n/a	AF298943	AF305282	•		LMF
	Ctenochromis horei	Lake Tanganyika	this study	C. Sturmbauer/W. Salzburger	AY929987	AY930100	•		Tr
	Ctenochromis oligacanthus	Congo River	Klett & Meyer 2002 [16]	n/a	-	AF416779	•		CSA
	Cyclopharynx fwae	Lake Fwa area, Congo Drainage	Salzburger et al. 2002 [9]	n/a	AF400711	-			CSA
	Cyclopharynx fwae	Lake Fwa area, Congo Drainage	this study	U. Schliewen	AY929986	AY930099	•		CSA
	Cyrtocara moorii	Lake Malawi	Lee et al. 1995 [80]	n/a	U12554	-			LMF
	Cyrtocara moorii	Lake Malawi	this study	I. Kornfield	AY929976	AY930089	•		LMF
	Cyrtocara moorii	Lake Malawi	this study	aquarium	AY930004	-			LMF
	Diplotaxodon greenwoodi	Lake Malawi	Shaw et al. 2000 [62]	n/a	AF298930	AF305269	•		LMF
	Diplotaxodon limnothrissa	Lake Malawi	Shaw et al. 2000 [62]	n/a	AF298922	AF305261	•		LMF

Haplochromis adolfifrederici	Lake Kivu	Verheyen et al. 2003 [4]	n/a	AY226715	-			LVS
Haplochromis astatodon	Lake Kivu	Verheyen et al. 2003 [4]	n/a	AY226650	-			LVS
Haplochromis astatodon	Lake Kivu	Verheyen et al. 2003 [4]	n/a	AY226669	-			LVS
Haplochromis astatodon	Lake Kivu	Verheyen et al. 2003 [4]	n/a	AY226631	-			LVS
Haplochromis astatodon	Lake Kivu	Verheyen et al. 2003 [4]	n/a	AY226697	-			LVS
Haplochromis astatodon	Lake Kivu	Verheyen et al. 2003 [4]	n/a	AY226651	-			LVS
Haplochromis astatodon	Lake Kivu	Verheyen et al. 2003 [4]	n/a	AY226632	-			LVS
Haplochromis astatodon	Lake Kivu	Verheyen et al. 2003 [4]	n/a	AY226654	-			LVS
Haplochromis astatodon	Lake Kivu	Verheyen et al. 2003 [4]	n/a	AY226692	-			LVS
Haplochromis astatodon	Lake Kivu	Verheyen et al. 2003 [4]	n/a	AY226652	-			LVS
Haplochromis astatodon	Lake Kivu	Verheyen et al. 2003 [4]	n/a	AY226691	-			LVS
Haplochromis bloyeti	Lukaware River, Kenyia	this study	L. De Vos (F2A-12/93)	AY929953	AY930058	•		EAR
Haplochromis crebridens	Lake Kivu	Verheyen et al. 2003 [4]	n/a	AY226714	-			LVS
Haplochromis gracilior	Lake Kivu	Verheyen et al. 2003 [4]	n/a	AY226790	-			EAR
Haplochromis gracilior	Lake Kivu	Verheyen et al. 2003 [4]/this study	E. Verheyen; K8	AY226788	AY930078	•		EAR
Haplochromis gracilior	Lake Kivu	Verheyen et al. 2003 [4]/this study	E. Verheyen; K9	AY226789	AY930079	•		EAR
Haplochromis graueri	Lake Kivu	Verheyen et al. 2003 [4]	n/a	AY226648	-			LVS
Haplochromis graueri	Lake Kivu	Verheyen et al. 2003 [4]	n/a	AY226668	-			LVS
Haplochromis graueri	Lake Kivu	Verheyen et al. 2003 [4]	n/a	AY226649	-			LVS
Haplochromis insidiae	Lake Kivu	Verheyen et al. 2003 [4]/this study	E. Verheyen	AY226675	AY930077	•		LVS
Haplochromis insidiae	Lake Kivu	Verheyen et al. 2003 [4]	n/a	AY226627	-			LVS
Haplochromis lividus	Lake Victoria	Nagl et al. 2000 [25]	n/a	AF213523	-			LVS
Haplochromis microchrysomelas	Lake Kivu	Verheyen et al. 2003 [4]	n/a	AY226695	-			LVS
Haplochromis nigroides/scheffersi	Lake Kivu	Verheyen et al. 2003 [4]	n/a	AY226629	-			LVS
Haplochromis obliquidens	Lake Victoria	this study	aquarium	AY929984	AY930097	•		LVS
Haplochromis occultidens	Lake Kivu	Verheyen et al. 2003 [4]	n/a	AY226666	-			LVS
Haplochromis paludinosus	Nanganga, Burundi	this study	L. De Vos (T2-5/27/93)	AY929994	AY930107	•		EAR
Haplochromis paucidens	Lake Kivu	Verheyen et al. 2003 [4]	n/a	AY226687	-			LVS
Haplochromis paucidens	Lake Kivu	Verheyen et al. 2003 [4]	n/a	AY226634	-			LVS
Haplochromis paucidens	Lake Kivu	Verheyen et al. 2003 [4]	n/a	AY226712	-			LVS
Haplochromis paucidens	Lake Kivu	Verheyen et al. 2003 [4]	n/a	AY226640	-			LVS
Haplochromis paucidens	Lake Kivu	Verheyen et al. 2003 [4]	n/a	AY226689	-			LVS
Haplochromis paucidens	Lake Kivu	Verheyen et al. 2003 [4]	n/a	AY226641	-			LVS
Haplochromis paucidens	Lake Kivu	Verheyen et al. 2003 [4]	n/a	AY226693	-			LVS
Haplochromis paucidens	Lake Kivu	Verheyen et al. 2003 [4]	n/a	AY226694	-			LVS
Haplochromis rubescens	Lake Kivu	Verheyen et al. 2003 [4]	n/a	AY226717	-			LVS
Haplochromis sauvagei	Lake Victoria (Kisumu, Kenya)	this study	L. De Vos (F2B-12/93)	AY929954	AY930059	•	II	LVS
Haplochromis sauvagei	Lake Victoria	this study	A. Meyer, T44	AY929958	AY930063	•	I	LVS
Haplochromis scheffersi	Lake Kivu	Verheyen et al. 2003 [4]	n/a	AY226647	-			LVS
Haplochromis scheffersi	Lake Kivu	Verheyen et al. 2003 [4]	n/a	AY226667	-			LVS
Haplochromis sp.	Lake Mburo, Uganda	Verheyen et al. 2003 [4]/this study	E. Schraml (9796)	AY226780	AY930082	•	II	LVS
Haplochromis sp.	Lake Ruvihinda, Burundi	this study	L. De Vos (T18-Aug 93)	AY930008	-			LVS
Haplochromis sp.	Lake Victoria	this study	A. Meyer (V7-Feb 93)	AY929960	AY930065	•	I	LVS
Haplochromis sp.	Lake Victoria	this study	A. Meyer (V12-Feb 93)	AY930009	-			LVS
Haplochromis sp.	Lake Victoria	this study	A. Meyer (V24-Feb 93)	AY930010	-			LVS
Haplochromis sp.	Lake Victoria	this study	A. Meyer (V25-Feb 93)	AY930011	-			LVS
Haplochromis sp.	Lake Victoria	this study	A. Meyer (V29-Feb 93)	AY930012	-			LVS
Haplochromis sp.	Lake Victoria Region (L. Kanyaboli)	this study	R. Abila (R612-2002)	AY930013	-			LVS
Haplochromis sp.	Lake Victoria Region (L. Kanyaboli)	this study	R. Abila (R613-2002)	AY930014	-			LVS
Haplochromis sp.	Tanzania	this study	L. Seegers (92/12)	AY930015	-			LVS
Haplochromis sp.	Tanzania	this study	L. Seegers (93/11B)	AY930016	-			EAR
Haplochromis sp.	Tanzania	this study	L. De Vos (H62)	AY929988	AY930101	•	Tanzania IV	LVS
Haplochromis sp.	Tanzania	this study	L. De Vos (H63)	AY929989	AY930102	•	Tanzania V	LVS
Haplochromis sp.	Tanzania	this study	L. Seegers (93/3)	AY929990	AY930103	•	Tanzania II	EAR
Haplochromis sp.	Tanzania	this study	L. Seegers (93/40)	AY929991	AY930104	•	Tanzania I	EAR
Haplochromis sp.	Tanzania	this study	L. Seegers (93/8)	AY929992	AY930105	•	Tanzania II	LVS
Haplochromis sp.	Upper Rusizi River, Burundi	this study	L. De Vos (T13-Aug 93)	AY929983	AY930096	•		LVS
Haplochromis sp.	Lower Rusizi River, Burundi	this study	L. De Vos (T9-Aug 93)	AY930017	-			LVS
Haplochromis sp.	Lake Victoria Region (L. Kanyaboli)	this study	R. Abila (R096-2002)	AY930018	-			LVS
Haplochromis sp.	Kazinga (Lakes Edward/George)	Nagl et al. 2000 [25]	n/a	AF213609	-			EAR

Haplochromis sp.	Lake Albert	Nagl et al. 2000 [25]	n/a	AF213573	-	LVS
Haplochromis sp.	Lake Albert	Nagl et al. 2000 [25]	n/a	AF213571	-	LVS
Haplochromis sp.	Lake Albert	Nagl et al. 2000 [25]	n/a	AF213574	-	LVS
Haplochromis sp.	Lake Albert	Nagl et al. 2000 [25]	n/a	AF213572	-	LVS
Haplochromis sp.	Lake Babati	Nagl et al. 2000 [25]	n/a	AF213606	-	EAR
Haplochromis sp.	Lake Bunyoni, Uganda	Verheyen et al. 2003 [4]	n/a	AY226773	-	LVS
Haplochromis sp.	Lake Bunyoni, Uganda	Verheyen et al. 2003 [4]	n/a	AY226784	-	LVS
Haplochromis sp.	Lake Chala, Tanzania	Nagl et al. 2000 [25]	n/a	AF213605	-	EAR
Haplochromis sp.	Lake Cohoha, Burundi	Verheyen et al. 2003 [4]	n/a	AY226725	-	LVS
Haplochromis sp.	Lake Cohoha, Burundi	Verheyen et al. 2003 [4]	n/a	AY226720	-	LVS
Haplochromis sp.	Lake Cohoha, Burundi	Verheyen et al. 2003 [4]	n/a	AY226726	-	LVS
Haplochromis sp.	Lake Cohoha, Burundi	Verheyen et al. 2003 [4]	n/a	AY226719	-	LVS
Haplochromis sp.	Lake Edward	Nagl et al. 2000 [25]	n/a	AF213564	-	LVS
Haplochromis sp.	Lake Edward	Nagl et al. 2000 [25]	n/a	AF213565	-	LVS
Haplochromis sp.	Lake Edward	Nagl et al. 2000 [25]	n/a	AF213569	-	LVS
Haplochromis sp.	Lake Edward	Nagl et al. 2000 [25]	n/a	AF213566	-	LVS
Haplochromis sp.	Lake Edward	Nagl et al. 2000 [25]	n/a	AF213577	-	LVS
Haplochromis sp.	Lake Edward	Nagl et al. 2000 [25]	n/a	AF213568	-	LVS
Haplochromis sp.	Lake Edward	Verheyen et al. 2003 [4]	n/a	AY226737	-	LVS
Haplochromis sp.	Lake Edward	Verheyen et al. 2003 [4]	n/a	AY226758	-	LVS
Haplochromis sp.	Lake George	Nagl et al. 2000 [25]	n/a	AF213611	-	EAR
Haplochromis sp.	Lake George	Nagl et al. 2000 [25]	n/a	AF213612	-	EAR
Haplochromis sp.	Lake George	Nagl et al. 2000 [25]	n/a	AF213610	-	EAR
Haplochromis sp.	Lake George	Nagl et al. 2000 [25]	n/a	AF213563	-	LVS
Haplochromis sp.	Lake George	Nagl et al. 2000 [25]	n/a	AF213559	-	LVS
Haplochromis sp.	Lake George	Nagl et al. 2000 [25]	n/a	AF213562	-	LVS
Haplochromis sp.	Lake George	Nagl et al. 2000 [25]	n/a	AF213560	-	LVS
Haplochromis sp.	Lake Manyara, Tanzania	Nagl et al. 2000 [25]	n/a	AF213607	-	EAR
Haplochromis sp.	Lake Mburo, Uganda	Verheyen et al. 2003 [4]	n/a	AY226749	-	LVS
Haplochromis sp.	Lake Mugogo, Uganda	Verheyen et al. 2003 [4]	n/a	AY226731	-	LVS
Haplochromis sp.	Lake Nawampasa, Uganda	Verheyen et al. 2003 [4]	n/a	AY226761	-	LVS
Haplochromis sp.	Lake Nawampasa, Uganda	Verheyen et al. 2003 [4]	n/a	AY226766	-	LVS
Haplochromis sp.	Lake Nyamusingire, Uganda	Verheyen et al. 2003 [4]	n/a	AY226755	-	LVS
Haplochromis sp.	Lake Victoria	Verheyen et al. 2003 [4]	n/a	AY226779	-	LVS
Haplochromis sp.	Lake Victoria	Verheyen et al. 2003 [4]	n/a	AY226762	-	LVS
Haplochromis sp.	Lake Victoria	Verheyen et al. 2003 [4]	n/a	AY226727	-	LVS
Haplochromis sp.	Lake Victoria	Verheyen et al. 2003 [4]	n/a	AY226767	-	LVS
Haplochromis sp.	Lake Victoria Region	Nagl et al. 2000 [25]	n/a	AF213588	-	LVS
Haplochromis sp.	Lake Victoria Region	Nagl et al. 2000 [25]	n/a	AF213582	-	LVS
Haplochromis sp.	Lake Victoria Region	Nagl et al. 2000 [25]	n/a	AF213583	-	LVS
Haplochromis sp.	Lake Victoria Region	Nagl et al. 2000 [25]	n/a	AF213581	-	LVS
Haplochromis sp.	Lakes Edward/George	Nagl et al. 2000 [25]	n/a	AF213557	-	LVS
Haplochromis sp.	Lakes Edward/George	Nagl et al. 2000 [25]	n/a	AF213561	-	LVS
Haplochromis sp.	Lakes Edward/George	Nagl et al. 2000 [25]	n/a	AF213558	-	LVS
Haplochromis sp.	Lakes Edward/George	Nagl et al. 2000 [25]	n/a	AF213566	-	LVS
Haplochromis sp.	Lakes Edward/George	Nagl et al. 2000 [25]	n/a	AF213584	-	LVS
Haplochromis sp.	Lakes Edward/George	Nagl et al. 2000 [25]	n/a	AF213570	-	LVS
Haplochromis sp.	Lakes Edward/George	Nagl et al. 2000 [25]	n/a	AF213586	-	LVS
Haplochromis sp.	Lakes Edward/George	Nagl et al. 2000 [25]	n/a	AF213585	-	LVS
Haplochromis sp.	Lakes Lutoto/Edward	Nagl et al. 2000 [25]	n/a	AF213567	-	LVS
Haplochromis sp.	Lakes Lutoto/Edward	Nagl et al. 2000 [25]	n/a	AF213575	-	LVS
Haplochromis sp.	Lakes Lutoto/Edward	Nagl et al. 2000 [25]	n/a	AF213576	-	LVS
Haplochromis sp.	Lakes Nshere/George	Nagl et al. 2000 [25]	n/a	AF213579	-	LVS
Haplochromis sp.	Lakes Nshere/George	Nagl et al. 2000 [25]	n/a	AF213587	-	LVS
Haplochromis sp.	Lakes Nshere/George	Nagl et al. 2000 [25]	n/a	AF213580	-	LVS
Haplochromis sp.	Lupa River, Tanzania	Nagl et al. 2000 [25]	n/a	AF213595	-	EAR
Haplochromis sp.	Malagarazi River	Nagl et al. 2000 [25]	n/a	AF213590	-	EAR
Haplochromis sp.	Malagarazi River	Nagl et al. 2000 [25]	n/a	AF213591	-	EAR
Haplochromis sp.	Malagarazi River	Nagl et al. 2000 [25]	n/a	AF213592	-	EAR
Haplochromis sp.	Malagarazi River	Nagl et al. 2000 [25]	n/a	AF213589	-	EAR

Haplochromis sp.	Malagarazi River	Nagl et al. 2000 [25]	n/a	AF213593	-		EAR
Haplochromis sp.	Malagarazi River	Nagl et al. 2000 [25]	n/a	AF213594	-		EAR
Haplochromis sp.	Malagarazi River	Nagl et al. 2000 [25]	n/a	AF213613	-		EAR
Haplochromis sp.	Malagarazi River	Nagl et al. 2000 [25]	n/a	AF213608	-		EAR
Haplochromis sp.	Malagarazi River	Nagl et al. 2000 [25]	n/a	AF213614	-		EAR
Haplochromis sp.	Myunga River, Tanzania	Nagl et al. 2000 [25]	n/a	AF213556	-		EAR
Haplochromis sp.	Pangani River, Tanzania	Nagl et al. 2000 [25]	n/a	AF213600	-		EAR
Haplochromis sp.	Pangani River, Tanzania	Nagl et al. 2000 [25]	n/a	AF213599	-		EAR
Haplochromis sp.	Pangani River, Tanzania	Nagl et al. 2000 [25]	n/a	AF213604	-		EAR
Haplochromis sp.	Piti River, Tanzania	Nagl et al. 2000 [25]	n/a	AF213598	-		EAR
Haplochromis sp.	Piti River, Tanzania	Nagl et al. 2000 [25]	n/a	AF213596	-		EAR
Haplochromis sp.	Piti River, Tanzania	Nagl et al. 2000 [25]	n/a	AF213597	-		EAR
Haplochromis sp.	Victoria Nile River	Verheyen et al. 2003 [4]	n/a	AY226734	-		LVS
Haplochromis sp.	Wogo River/Lake Rukwa	Nagl et al. 2000 [25]	n/a	AF213602	-		EAR
Haplochromis sp.	Wogo River/Lake Rukwa	Nagl et al. 2000 [25]	n/a	AF213603	-		EAR
Haplochromis sp.	Wogo River/Lake Rukwa	Nagl et al. 2000 [25]	n/a	AF213601	-		EAR
Haplochromis sp.	Wogo River/Lake Rukwa	Nagl et al. 2000 [25]	n/a	AF213555	-		EAR
Haplochromis sp. "Dwarf Big Eye"	Lake Victoria Region (L. Kanyaboli)	this study	R. Abila (R280-2002)	AY929968	AY930074	•	LVS
Haplochromis sp. "Dwarf Big Eye"	Lake Victoria Region (L. Kanyaboli)	this study	R. Abila (R193-2002)	AY930042	-		LVS
Haplochromis sp. "Dwarf Big Eye"	Lake Victoria Region (L. Kanyaboli)	this study	R. Abila (R048-2002)	AY930043	-		LVS
Haplochromis sp. "oblique mouth"	Lake Cohoha, Burundi	this study	L. De Vos (T10-Aug 93)	AY930041	-		LVS
Haplochromis sp. "rockkribensis"	Lake Victoria	this study	O. Seehausen	AY390039	-		LVS
Haplochromis sp. "rockkribensis"	Lake Victoria	this study	O. Seehausen	AY390040	-		LVS
Haplochromis sp. "rockkribensis"	Lake Victoria	Nagl et al. 2000 [25]	n/a	AF213541	-		LVS
Haplochromis sp. "rockkribensis"	Lake Victoria	Nagl et al. 2000 [25]	n/a	AF213554	-		LVS
Haplochromis sp. "rockkribensis"	Lake Victoria	Nagl et al. 2000 [25]	n/a	AF213542	-		LVS
Haplochromis sp. "small red"	Lake Cohoha, Burundi	this study	L. De Vos (T11-Aug 93)	AY930036	-		LVS
Haplochromis sp. "small red"	Lake Rweru, Burundi	this study	L. De Vos (T16-Aug 93)	AY930038	-		LVS
Haplochromis sp. "thick lips"	Lake Cohoha, Burundi	this study	L. De Vos (T12-93)	AY930037	-		LVS
Haplochromis sp. crebridens/olivaceus	Lake Kivu	Verheyen et al. 2003 [4]	n/a	AY226623	-		LVS
Haplochromis sp. crebridens/olivaceus	Lake Kivu	Verheyen et al. 2003 [4]	n/a	AY226704	-		LVS
Haplochromis sp. crebridens/olivaceus	Lake Kivu	Verheyen et al. 2003 [4]	n/a	AY226646	-		LVS
Haplochromis sp. crebridens/olivaceus	Lake Kivu	Verheyen et al. 2003 [4]	n/a	AY226645	-		LVS
Haplochromis sp. crebridens/olivaceus	Lake Kivu	Verheyen et al. 2003 [4]	n/a	AY226642	-		LVS
Haplochromis sp. crebridens/olivaceus	Lake Kivu	Verheyen et al. 2003 [4]	n/a	AY226670	-		LVS
Haplochromis sp. nov	Kisangani (Lualaba River), DR Congo	this study	L. De Vos (6/13/95)	AY929957	AY930062	•	CSA
Haplochromis sp. velvet black	Lake Victoria	Nagl et al. 2000 [25]	n/a	AF213543	-		LVS
Haplochromis squamipinnis	Lake Edward	Verheyen et al. 2003 [4]/this study	E. Schraml (9813)	AY226747	AY930083	•	LVS
Haplochromis stappersi	Malagarazi River	this study	L. De Vos (5-6/25/92)	AY929941	AY930046	•	LVS
Labeotropheus trevawasae	Lake Malawi	Nagl et al. 2000 [25]	n/a	AF213623	-		LMF
Lethrinops auritus	Lake Malawi	Lee et al. 1995/Kocher et al. 1995 [15]	n/a	U12551	U07252	•	LMF
Lethrinops furcifer	Lake Malawi	Shaw et al. 2000 [62]	n/a	AF298977	AF305316	•	LMF
Lethrinops longipinnis	Lake Malawi	Shaw et al. 2000 [62]	n/a	AF298956	AF305296	•	LMF
Lipochromis maxillaris	Lake Victoria Region (L. Kanyaboli)	this study	R. Abila (R542-2002)	AY930034	-		LVS
Lipochromis melanopterus	Lake Victoria	Nagl et al. 2000 [25]	n/a	AF213527	-		LVS
Macropleurodus bicolor	Lake Victoria	this study	A. Meyer (Mb2-Feb 33); T47	AY930007	-		LVS
Melanochromis auratus	Lake Malawi	this study	aquarium	AY929964	AY930069	•	LMF
Neochromis nigricans	Lake Victoria	Nagl et al. 2000 [25]	n/a	AF213528	-		LVS
Neochromis nigricans	Lake Victoria	Nagl et al. 2000 [25]	n/a	AF213545	-		LVS
Neochromis nigricans	Lake Victoria	Nagl et al. 2000 [25]	n/a	AF213544	-		LVS
Neochromis nigricans	Lake Victoria (Makobe Island)	this study	O. Seehausen (Ma 8/1-Dec. 93)	AY930006	-		LVS
Neochromis nigricans	Lake Victoria (Nane Island)	this study	L. De Vos (F2C-12/93)	AY930005	-		LVS
Orthochromis kasuluensis	Mgandazi River, Tanzania	this study	L. De Vos (4C)	AY930031	-		MO
Orthochromis kasuluensis	Tanzania	this study	L. De Vos (T2-July 94)	AY929944	AY930049		MO
Orthochromis luichensis	Mkuti River, Luiche Basin, Tanzania	this study	L. De Vos (T94/3)	AY929947	AY930052	•	MO
Orthochromis malagaraziensis	Malagarazi River	Salzburger et al. 2002 [9]	n/a	AF400714	AF398232	•	MO
Orthochromis malagaraziensis	Nyarungunga River, Burundi	this study	L. De Vos (7-2/19/93)	AY929949	AY930054	•	MO
Orthochromis malagaraziensis	Nyarungunga River, Burundi	this study	L. De Vos (T5-5/28/93)	AY929951	AY930056	•	MO
Orthochromis mazimeroensis	Nanganga, Burundi	this study	L. De Vos (T1-5/27/93)	AY930032	-		MO
Orthochromis mazimeroensis	Mazimero River, Burundi	this study	L. De Vos (T3-5/27/93)	AY929948	AY930053	•	MO

	Orthochromis mosoensis	Ruisseau Gytinya, Burundi	this study	L. De Vos (T7-5/28/93)	AY930033	-	•		MO
	Orthochromis mosoensis	Ruisseau Gytinya, Burundi	this study	L. De Vos (T8-5/28/93)	AY929950	AY930055			MO
	Orthochromis polyacanthus	Congo River	Salzburger et al. 2002 [9]	n/a	AF400712	AF398231	•		CSA
	Orthochromis rubrolabialis	Tanzania	this study	L. Seegers (TZ94-108)	AY929946	AY930051	•		MO
	Orthochromis rugufuensis	Rugufu River, Tanzania	this study	L. Seegers (TZ94-121)	AY929945	AY930050	•		MO
	Orthochromis uvinzae	Malagarazi River	this study	L. Seegers (TZ94-112b)	AY929943	AY930048	•		MO
	Paralabidochromis beadlei	Lake Victoria	Nagl et al. 2000 [25]	n/a	AF213519	-			LVS
	Paralabidochromis chilotes	Lake Victoria	Nagl et al. 2000 [25]	n/a	AF213540	-			LVS
	Paralabidochromis chilotes	Lake Victoria	Nagl et al. 2000 [25]	n/a	AF213525	-			LVS
	Paralabidochromis chilotes	Lake Victoria	Nagl et al. 2000 [25]	n/a	AF213539	-			LVS
	Paralabidochromis plagiodon	Lake Victoria	Nagl et al. 2000 [25]	n/a	AF213546	-			LVS
	Paralabidochromis plagiodon	Lake Victoria	Nagl et al. 2000 [25]	n/a	AF213529	-			LVS
	Paralabidochromis plagiodon	Lake Victoria	Nagl et al. 2000 [25]	n/a	AF213547	-			LVS
	Pharyngochromis acuticeps	Zambezi River, Zambia	this study	C. Katongo/C. Sturmbauer	AY929981	AY930094	•		CSA
	Platytaeniodus degeni	Lake Victoria	this study	A. Meyer (Pd1)	AY929959	AY930064	•		LVS
	Platytaeniodus degeni	Lake Victoria	this study	A. Meyer (Pd2)	AY930030	-			LVS
	Prognathochromis venator	Lake Victoria	Nagl et al. 2000 [25]	n/a	AF213537	-			LVS
	Psammochromis riponianus	Lake Victoria	Nagl et al. 2000 [25]	n/a	AF213530	-			LVS
	Pseudocranilabrus multicolor I	Lake Victoria Region	Salzburger et al. 2002 [9]	n/a	AF400713	AF398233			LVS
	Pseudocranilabrus multicolor V	Lake Victoria Region (L. Kanyaboli)	this study	R. Abila (R082-2002)	AY929965	AY930070	•	victoriae	Ps
	Pseudocranilabrus multicolor VII	Lake Victoria	this study	A. Meyer; T31	AY930029	-			LVS
	Pseudocrenilabrus multicolor	Tanzania	this study	L. Seegers (91/137)	AY929993	AY930106	•		Ps
	Pseudocrenilabrus multicolor III	Lake Victoria Region (L. Kanyaboli)	this study	R. Abila (R084-2002)	AY930028	-			Ps
	Pseudocrenilabrus multicolor IV	Lake Victoria Region (L. Kanyaboli)	this study	R. Abila (R083-2002)	AY930027	-			Ps
	Pseudocrenilabrus philander	Zambezi River	this study	aquarium	AY929942	AY930047	•		Ps
	Pseudocrenilaburs multicolor VI	Lake Victoria Region (L. Kanyaboli)	this study	R. Abila (R081-2002)	AY930026	-			Ps
	Pseudotropheus livingstonii	Lake Malawi	this study	I. Kornfield	AY929956	AY930061	•		LMF
	Pseudotropheus msobo	Lake Malawi	Nagl et al. 2000 [25]	n/a	AF213622	-			LMF
	Pseudotropheus zebra	Lake Malawi	this study	aquarium	AY930025	-			LMF
	Pseudotropheus zebra "patricky"	Lake Malawi	this study	aquarium	AY930024	-			LMF
	Ptyochromis sauvagei	Lake Victoria	Nagl et al. 2000 [25]	n/a	AF213549	-			LVS
	Ptyochromis sauvagei	Lake Victoria	Nagl et al. 2000 [25]	n/a	AF213531	-			LVS
	Ptyochromis xenognathus	Lake Victoria	Nagl et al. 2000 [25]	n/a	AF213532	-			LVS
	Ptyochromis xenognathus	Lake Victoria	Nagl et al. 2000 [25]	n/a	AF213535	-			LVS
	Ptyochromis xenognathus	Lake Victoria	Nagl et al. 2000 [25]	n/a	AF213533	-			LVS
	Ptyochromis xenognathus	Lake Victoria	Nagl et al. 2000 [25]	n/a	AF213534	-			LVS
	Ptyochromis xenognathus	Lake Victoria	this study	A. Meyer (2-93)	AY930035	-			LVS
	Rhamphochromis esox	Lake Malawi	Shaw et al. 2000 [62]	n/a	AF298913	AF305252	•		LMF
	Rhamphochromis macrophthalmus	Lake Malawi	Shaw et al. 2000 [62]	n/a	AF298911	AF305250	•		LMF
	Sargochromis giardi	Zambezi River, Zambia	this study	C. Katongo/C. Sturmbauer (2647)	AY929985	AY930098	•		CSA
	Schwetzochromis stormsi	Kisangani (Lualaba River), DR Congo	this study	L. De Vos (5/5/95)	AY929952	AY930057	•		CSA
	Serranochromis sp.	Lake Mweru-Wantipa, Zambia	Verheyen et al. 2003 [4]/this study	T. Reuter/E. Schraml (9793)	AY226792	AY930081	•		CSA
	Thoracochromis brauschi	Lake Fwa area, Congo Drainage	this study	aquarium	AY929982	AY930095	•		CSA
	Thoracochromis brauschi	Lake Fwa area, Congo Drainage	this study	R. Paul/E. Schraml (9792)	AY226791	AY930080	•		CSA
	Tyrannochromis nigriventer	Lake Malawi	Shaw et al. 2000 [62]	n/a	AF298968	AF305307	•		LMF
	Xystichromis phytophagus	Lake Victoria Region (L. Kanyaboli)	this study	R. Abila (R670-2002)	AY929970	AY930076	•		LVS
	Yssichromis laparogramma	Lake Victoria	Nagl et al. 2000 [25]	n/a	AF213520	-			LVS
	Yssichromis laparogramma	Lake Victoria	Nagl et al. 2000 [25]	n/a	AF213521	-			LVS
Lamprologini	*Julidochromis marlieri*	Lake Tanganyika	Salzburger et al. 2002 [9]	n/a	AF400717	AF398230	•		LT
	Lamprologus callipterus	Lake Tanganyika	Salzburger et al. 2002 [9]	n/a	AF400718	AF398226	•		LT
	Lamprologus mocquardi	Congo River	Salzburger et al. 2002 [9]	n/a	AF400720	AF398225	•		LT
	Neolamprologus brichardi	Lake Tanganyika	Salzburger et al. 2002 [9]	n/a	AF400721	AF398227	•		LT
	Telmatochromis bifrenatus	Lake Tanganyika	Salzburger et al. 2002 [9]	n/a	AF400724	AF398228	•		LT
Limnochromini	*Gnathochromis pfefferi*	Lake Tanganyika	Salzburger et al. 2002 [9]/Kocher et al. 1995 [15]	n/a	AF400727	U07248	•		LT
	Limnochromis auritus	Lake Tanganyika	Salzburger et al. 2002 [9]	n/a	AF400728	AF398246	•		LT
	Triglachromis otostigma	Lake Tanganyika	Salzburger et al. 2002 [9]	n/a	AF400729	AF398217	•		LT
Perissodini	*Perissodus microlepis*	Lake Tanganyika	Salzburger et al. 2002 [9]	n/a	AF400730	AF398222	•		LT
	Plecodus straeleni	Lake Tanganyika	Salzburger et al. 2002 [9]	n/a	AF400731	AF398221	•		LT
Tilapiini	*Boulengerochromis microlepis*	Lake Tanganyika	this study/Klett & Meyer 2002 [16]	n/a	AY929939	AF317229	•		LT
	Oreochromis tanganicae	Lake Tanganyika	this study/Salzburger et al. 2002 [9]	n/a	AY929940	AF317240	•		LT

Tropheini	*Cyphotilapia frontosa*	Lake Tanganyika	Salzburger et al. 2002 [9]/Kocher et al. 1995 [15]	n/a	AF400732	U07247	•		Tr
	Lobochilotes labiatus	Lake Tanganyika	Salzburger et al. 2002 [9]/Kocher et al. 1995 [15]	n/a	AF400733	U07254	•		Tr
	Petrochromis macrognathus	Lake Tanganyika	this study	J. Snoeks	AY929963	AY930068	•		Tr
	Petrochromis orthognathus	Lake Tanganyika	this study	E. Verheyen	AY930023	-			Tr
	Simochromis diagramma	Lake Tanganyika	this study	E. Verheyen	AY929974	AY930087	•		Tr
	Simochromis marginatus	Lake Tanganyika	this study	E. Verheyen	AY929975	AY930088	•		Tr
	Tropheus brichardi	Lake Tanganyika	this study	E. Verheyen; M85	AY929973	AY930086			Tr
	Tropheus brichardi	Lake Tanganyika	this study	E. Verheyen; M87	AY930022	-	•		Tr
	Tropheus duboisi	Lake Tanganyika	this study	E. Verheyen; M7	AY929972	AY930085			Tr
	Tropheus duboisi	Lake Tanganyika	this study	E. Verheyen; M9	AY930021	-	•		Tr
	Tropheus moorii I	Lake Tanganyika	this study	E. Verheyen; T66	AY929961	AY930066	•	I	Tr
	Tropheus moorii II	Lake Tanganyika	this study	E. Verheyen; T67	AY929962	AY930067	•	II	Tr
	Tropheus moorii III	Lake Tanganyika	this study	E. Verheyen; 97	AY929978	AY930091	•	III	Tr
	Tropheus moorii IV	Lake Tanganyika	this study	E. Verheyen; 116	AY929980	AY930093	•	IV	Tr
	Tropheus moorii V	Lake Tanganyika	this study	E. Verheyen; 93	AY930020	-			Tr
	Tropheus polli	Lake Tanganyika	this study	E. Verheyen	AY929971	AY930084	•		Tr
	Tropheus sp.	Lake Tanganyika	this study	E. Verheyen	AY930019	-			Tr
